# Freeze-Dried Powder of Fermented Chili Paste—New Approach to Cured Salami Production

**DOI:** 10.3390/foods11223716

**Published:** 2022-11-18

**Authors:** Adriana Păucean, Csaba Balázs Kádár, Elemér Simon, Dan Cristian Vodnar, Floricuța Ranga, Iulian Eugen Rusu, Vasile-Gheorghe Vișan, Sonia-Ancuța Socaci, Simona Man, Maria Simona Chiș, Anamaria Pop, Anda E. Tanislav, Vlad Mureșan

**Affiliations:** 1 Department of Food Engineering, Faculty of Food Science and Technology, University of Agricultural Sciences and Veterinary Medicine, 3–5 Mănăștur Street, 400372 Cluj-Napoca, Romania; 2 Department of Food Science, Faculty of Food Science and Technology, University of Agricultural Sciences and Veterinary Medicine, 3–5 Calea Mănăstur, 400372 Cluj-Napoca, Romania; 3 Department of Fundamental Sciences, Faculty of Animal Science and Biotechnologies, University of Agricultural Sciences and Veterinary Medicine, 3–5 Mănăștur Street, 400372 Cluj-Napoca, Romania

**Keywords:** fermented chili, bio-molecules, cured meat products, lactic acid bacteria, acidity, preservation

## Abstract

Fermented chili powders were obtained through the freeze-drying of fermented chili pastes and used as a condiment, acidifier, antioxidant, colorant, and microbial starter carrier in fermented salami production. Fermented chili powders were examined regarding carbohydrates, organic acids, vitamin C, phenolic compounds, carotenoids, and aroma profile. High concentrations of lactic (10.57–12.20%) and acetic acids (3.39–4.10%) were recorded. Vitamin C content was identified in the range of 398–1107 mg/100 g, with maximum values for *C. annuum* cv. Cayenne chili powder. Phenolic compounds showed values between 302–771 mg/100 g. Total carotenoid content was identified between 544–2462 µg/g, with high concentrations of capsanthin esters. Aroma profile analysis evidenced specific compounds (1-hexanol, 2-hexanol, hexenal, E-2-hexenal) with sensory importance and a more complex spectrum for *Capsicum chinense* cultivar. Plant-specific lactic acid bacteria showed dominance both in fermented chili paste, chili powder, and salami. Lactic and acetic acids from the fermented chili powder reduced the pH of the filling immediately, having a stabilizing effect on the meat. Nor molds or pathogens were identified in outer limits. Based on these results, fermented chili powders could be used as starter carriers in the production of fermented meat products for exceptional sensory properties and food safety management.

## 1. Introduction

Conventional chili powder is obtained from air-dried chili pepper fruits and it is used as a spice/condiment in a great variety of dishes [1]. It is also greatly appreciated and explored in the production of many types of meat products, especially fermented/cured sausages, salami, etc. [2,3]. Dried pepper powders are rarely contaminated by pathogen bacteria, although contaminations are not excluded. The European Union does not require the microbiological examination of herbs and spices [4]. Studies suggest that in some cases total viable counts in different paprika samples might reach values of 10^6^ CFU/g, whereas yeasts and molds occur in lower concentrations (<10^3^ CFC/g) [4]. 

Contaminations with toxins produced by fungi during the growth and/or storage of chili/pepper fruits after harvest do appear [5], representing a great technological and health problem [6,7]. A study conducted on different chili powder samples showed that 96.7% of the examined samples were contaminated by fungi at concentrations of <10^3^ CFU/g [8]. The presence of both *Aspergillus flavus* and *Aspergillus niger* was also confirmed in numerous dried chili and chili powder samples at values of >10^3^ CFU/g [7]. Scientific observations confirm that most spices based on chili are contaminated both with molds and toxins, many samples exceeding the EU regulatory limit (20 µg/kg) for ochratoxin A [9]. Contamination is usually associated with high humidity and extended processing time [6]. For instance, mold development is usually conditioned by moisture content >15% [10]. In this regard, the quality of the raw material, humidity, and processing time is of key importance in the production of high-quality chili powders [8]. The fermentation of chilis can also stabilize chili pastes, as lactic acid bacteria (LAB) can outnumber less competitive microbes, especially pathogens [11]. Fermenting microorganisms (LAB) can also metabolize different toxins [12], thus leading the way for new technological approaches in chili preservation [13,14].

Besides microbiological safety, chili or paprika powder is often appreciated by the intensity of its color, exceptional aroma, taste, and high phenolic and vitamin C content [15,16]. It is generally accepted that chili powders originated from warm climate regions (Spain, Peru, etc.) and contain the highest amounts of carotenoids, thus also having the most intense color [4]. The production of high-quality paprika/chili powder with high vitamin C content implies the reduction of time exposure to thermal dehydration and the usage of low drying temperatures [17]. The preservation of color compounds (expanding shelf-life) can also be achieved by low thermal exposure [18] or by the addition of natural antioxidants (rosemary extract) or by the inclusion of seeds in the grinding process [16], which increases the antioxidant potential of the spice.

Chili/paprika powder was shown to exert protective effects against rancidity in dry sausage, correlated with low malonaldehyde content [19]. Capsaicinoids and phenolic compounds were identified as antimicrobial agents found in chilis, which might account for the preserving effect of chili spices incorporated into meat products [20,21].

Beneficial bacterial activity (LAB) represents a key element in the preservation of many food items, including meat, dairy, vegetables, etc. [22]. Even before scientific observations were carried out, spontaneous microflora, including yeasts [23] and molds, were unwillingly used to obtain and preserve fermented meat products [24,25], thus leading these microorganisms to play a critical role in modern food production and food safety [3,26]. LAB and coagulase-negative cocci play the main role in processes related to meat fermentation [22]. In spontaneously fermented sausages *Lactobacillus* ssp., *Weissella* ssp. and *Pediococcus* ssp. were found to be the predominant microorganisms with fluctuations during the process [27]. *Lactobacillus* ssp. are represented by *L. sakei*, *L. curvatus*, and *L. plantarum*, whereas coagulase-negative cocci are represented by *Staphylococcus xylosus* and *S. simulans* [3,22,28]. Yeasts are also found in fermented meat products, the most dominant species in Italian-type products being *Debaryomyces hansenii* [22,29], which in some cases can exert anti-mold effect on the surface of the product [30].

Studies have revealed that *staphylococci* are to be found in the outer layers of fermented salami-type products as a result of higher oxygen concentrations [31], whereas LAB proliferates in the low oxygen parts (core) [27]. The preserving effect (the inhibition of pathogen growth) of beneficial microorganisms in fermented meat products is due to the combination of sugar metabolism, organic acid synthesis, and the acidification of the substrate [25,28]. This leads to the lowering of pH values to optimum safety levels (pH < 5.2), which is usually achieved 48–72 h after incubation [22]. The accumulation of different metabolites during the fermentation and aging period leads to the formation (synthesis) of volatile (aroma) compounds, thus delivering exceptional sensorial properties to the final product [32].

Pathogen growth inhibition is strongly correlated with lactic and acetic acid synthesis [25,33]. Weak acids can penetrate the cell walls of different undesired bacteria, dissociate in the cytoplasm, and shift the metabolic balance of the cell, thus inhibiting its growth [34,35]. Usually, the preserving effect of acids is correlated with other processes, including salting, curing, aging, dehydration, spicing, etc., that can further stabilize the products through complex physical and chemical interactions, including the reduction of water activity, oxygen elimination, suppressing cell functions, etc. [14,19,36]. The use of nitrites/nitrates in meat products is also of critical importance for the inhibition of *Clostridium botulinum* [2].

The initial bacterial load of raw material (meat) is strongly associated with good hygienic practices and generally presents strong fluctuations (3.2–5.3 log CFU/g) [31]. Achieving the stability of the product requires a minimum ratio of 10:1 (desired and undesired) bacteria [35], which guarantees good competitiveness for fermenting bacteria [31]. These values are achieved by the incorporation of pure starter cultures in the manufacturing process.

Starter cultures were introduced as a necessity to have better control over food items, as food safety could not be taken for granted in fermented meat-type products [37]. Over the years, many episodes of food poisoning were caused by unpasteurized milk and contaminated meat, usually related to *Staphylococcus aureus* [38], *Escherichia coli*, *Salmonella* [39], and *Listeria monocytogenes* [40], etc. In the case of dairy products, the pasteurization of the milk was the answer to food safety, although compromises have been made in the sensorial properties of cheeses that previously relied on the endogenous microflora of the fresh milk. Vegetables were less susceptible to food poisoning, as pathogens are usually not adapted to these substrate conditions. Meat on the other hand, especially raw meat used in the production of fermented products, is highly susceptible to contamination [2,24]. As meat cannot be pasteurized or sterilized, contamination during production is reduced by selecting premium quality (fresh) raw material and by the addition of highly concentrated pure bacterial strains (starter cultures) [36,41] that can adapt quickly, proliferate and overtake (numerically) the substrate in the shortest time possible, under the guidance of well-controlled physical parameters (temperature, humidity, airspeed). Further prevention was taken with a high level of hygienic safety and quality monitoring during the entire process [37].

A new trend has been set in meat processing that reaches out to alternative systems and techniques that involve the usage of new bacterial strains, which might benefit consumer health [37] or the incorporation of fresh and fermented vegetables in meat products, which might increase their biological and sensorial quality [42].

Difficulties regarding capsicum production and processing (especially drying), are physically laborious and expensive. Usually, these operations imply the exposure of the raw material to bio-active compound losses through thermal stress and the possibility of mold contaminations (toxin producing agents). In this regard, it is desirable to reduce contamination risks and the thermal losses of bio-active agents. Fermentation and freeze-drying might represent an alternative to traditional processes or at least a complementary solution for high quality powder production. This might be achieved by the inclusion of fermenting activities in chili processing and the application of freeze-drying methods for rapid dehydration. The obtained powder (fermentate) could not only resemble the visual and textural properties of traditional chili powder but might also present enhanced biochemical (acidifier, antioxidant, antimicrobial), microbial (high counts of LAB), and sensorial (exceptional flavor and aroma) properties, which might further be explored in food technology (cured meat products). 

In this context, this study first aimed to analyze flavor compounds in different stages of processing (fresh and fermented) and to characterize the fermented chili powder regarding its content in different bio-compounds (carotenoids, phenolic compounds, ascorbic acid, carbohydrates, and carboxylic compounds). 

The second objective was to analyze the traceability of plant-specific microflora during the different stages of chili processing and to assess the microbial adaptability and growth on protein substrate (meat) during the production of fermented salami, as presented in the Graphical Abstract.

## 2. Materials and Methods

### 2.1. Preparation of Fermented Chili (Freeze-Dried) Powder

The different cultivars of *Capsicum annuum* (*C. annuum* cv. Cherry, *C. annuum* cv. Cayenne), and *Capsicum chinense* (*C. chinense* cv. Fatalii, *C. chinense* cv. Habanero) chili samples were acquired from a local producer at fully matured (ripened) stage. Fruits were washed, halved (peduncle discarded), and blended (Robot Cook 3.7 l., Robot Coupe, Vincennes, France) in order to be fermented. No salt, spices, or additives were added to the mash. Fresh chili paste was spontaneously fermented for 21 days at 20 ± 1°C. 

Fermented chili paste was freeze-dried (−55 °C; 72 h; *p* = 0.001 mbar) with a laboratory freeze-dryer (Telstar Lyo Quest 55 plus) to obtain fermented chili powder.

I. Determinations for fermented chili paste and freeze-dried chili powder

### 2.2. Carotenoidic Compounds Analysis

Carotenoids were analized in accordance with similar studies described in the literature [43] with some adjustments.

A total 0.5 g of each freeze-dried fermented chili powder sample was mixed with the solvent (1/1/1; *v*/*v*/*v*) consisting of three components (methanol/ethyl acetate/petroleum ether). Samples were vortexed (1 min) using a Heidolph Reax vortex (Sigma-Aldrich Chemie GmbH, Taufkirchen, Germany, sonicated (Elmasonic E 15 H), and centrifuged at 7155 g for 10 min. (Eppendorf AG 5804). The process was repeated 7–8 times on each sample until total extraction (complete discoloration of each chili powder sample). Extracts were cumulated, filtered, and washed with saturated NaCl solution (30%). The organic phase was filtered through anhydrous Na_2_SO_4_. In the end, petroleum ether was pipetted on the surface of the filter to carry through all remaining carotenoids. The extract was subdued to evaporation at low temperature (40 °C) using a rotative evaporator (Heidolph Hei-VAP Expert). Each sample was redissolved in 1 mL of ethyl-acetate, filtered (Chromafil Xtra nylon; 0.45 μm), and injected into the HPLC system.

HPLC analysis was carried out by the HPLC Agilent 1200 system equipped with a quaternary pump and autosampler and UV–Vis coupled with photodiode (DAD), Agilent Technologies, Santa Clara, CA, USA. For the separation of the compounds the EC 250/4.6 Nucleodur 300-5 C-18, etc., (250 × 4.6 mm, 5 µm) column was used (Macherey-Nagel, Düren, Germany) with the temperature settled at 25 °C.

The mobile phase conceived of acetonitrile/water/triethylamine solution 90/10/0.25 (A) and ethyl-acetate/triethylamine 100/0.25 (B) with the following gradient: min 0, 90% A; min 10, 50% A. Solvent A decreased from 50% in the first minute to 10% after 10 min. The flow rate was 1 mL/min. Chromatograms were registered at λ = 450 nm.

Capsanthin, zeaxanthin, and β-carotene standards (99% purity) were acquired (Sigma-Aldrich, Burlington, MA, USA) for the identification and quantification of carotenoids in fermented chili powder samples. Five different concentrations were injected three times in the HPLC system. 

Esters of different carotenoids were identified based on literature.

### 2.3. Phenolic Compounds Analysis

The extraction and quantification of phenolic compounds was based on recently conducted, similar studies [44], with some moderate changes.

A total of 25 mL methanol and 1% HCl acid were added to 0.1 g of each freeze-dried chili powder sample. The test tubes were vortexed (Heidoph Reax) and sonicated for 30 min (Elmasonic E 15 H), macerated for 24 h at 4 °C, and centrifuged at 7155 g for 10 min at room temperature (Eppendorf AG 5804). The supernatant of each freeze-dried chili sample was filtered (Chromafil Xtra nylon; 0.45 μm). A total 20 μL of extract were injected into the HPLC system. Chromatograms were registered at λ = 340 nm for the examined compounds. 

For the quantification of phenolic compounds (hydroxybenzoic acids, flavanols, flavanones) calibration was made carried out, using five different concentrations of gallic acid, as for flavones and flavonols, calibration was made with rutin. Standards were acquired from Sigma-Aldrich. HPLC analysis was carried out using the Agilent 1200 system equipped with a quaternary pump, autosampler, and UV–Vis coupled with diode-array detection (DAD) and mass detector (MS) Agilent 6110 (Agilent Technologies, Santa Clara, CA, USA) were used. The same column was used as in the case of ascorbic acid, mobile phases consisting of A (H_2_O + 0.1% acetic acid) and B (acetonitrile + 0.1% acetic acid). Gradients (% B) were as presented: 0 min, 5% B; 0–2 min, 5% B; 2–18 min, 5–40% B; 18–20 min, 40–90% B; 20–24 min, 90% B; 24–25 min, 90–5% B; and 25–30 min, 5% B.

For mass spectra, electrospray ionization (ESI) technique was used and adapted to the following parameters: capillary voltage (3000 V), temperature (350 °C), nitrogen flow rate (7 L/min), and *m*/*z* (120–1200, full-scan). The interpretation of results was carried out by Agilent ChemStation software, B 02.01 SR2 version.

### 2.4. Ascorbic Acid Quantification

The protocol was based on recent studies conducted on different chili samples with some moderate changes [45]. An amount of 0.1 g of freeze-dried chili powder from each sample was mixed with 5 mL aqueous solution of metaphosphoric acid (3%) and acetic acid (8%). Test tubes were vortexed for 1 min (Heidoph Reax), sonicated for 30 min, (Elmasonic E 15 H) and centrifuged (7155 g, at 4 °C) using an Eppendorf AG 5804 centrifuge. The supernatant of each chili sample was filtered (Chromafil Xtra nylon 0.45 µm) and 20 µL were injected into the HPLC system. The identification of the compounds was based on retention time, UV–Vis, and mass spectra. Retention times were identified at 3.15 min (ascorbic acid) and 4.08 min (dehydroascorbic acid).

Five different concentrations of standard solutions (99% purity) were injected three times into the HPLC system (calibration curve) for the quantification of the compounds. Standard solutions were acquired from Sigma-Aldrich, USA. HPLC analysis was carried out by using the HPLC Agilent 1200 system equipped with a quaternary pump, autosampler, and UV–Vis coupled with photodiode (DAD) and mass detector (MS) Agilent 6110 (Agilent Technologies, CA, USA). For the separation of the compounds, an Eclipse XDB C18 column was used (size: 4.6 × 150 mm). The mobile phase conceived of water/acetonitrile solution 95/5 (*v*/*v*) and 1% formic acid. The time flow rate was 0.5 mL/min for 10 min at 25 °C. Spectral values were registered at wavelengths ranging between 200–400 nm. Chromatograms were identified at λ = 240 nm. Electrospray ionization (ESI) technique was used with the following parameters: capillary voltage (3000 V), temperature (300 °C), and nitrogen flow rate (7 L/min), *m*/*z* (100–600, full-scan). The interpretation of the results was carried out by the Agilent ChemStation software, B 02.01 SR2 version.

### 2.5. Sugars and Acids Analysis

The experiment was based on the recent literature [45], with some moderate changes. A total of 1 g of each of freeze-dried chili powder sample was extracted with 4 mL of distilled water. Each test tube was vortexed for 1 min (Heidoph Reax), sonicated for 30 min. (Elmasonic E 15 H), and centrifuged (7155 g at 24 °C) using an Eppendorf AG 5804 centrifuge. The supernatant of each extract was filtered (Chromafil Xtra nylon 0.45 µm) and 20 µL were injected in the HPLC system

The separation and quantification of the compounds were carried out by the HPLC Agilent 1200 system, equipped with a quaternary pump, vent for solvents, and a manual injector coupled with RID (Refractive Index Detector) (Agilent Technologies, Santa Clara, CA, USA). 

For the separation of compounds, a Polaris Hi-Plex H 300 × 7.7 mm column was used (Agilent Techologies, Santa Clara, CA, USA). The mobile phase comprised H_2_SO_4_ 5 mM solution with a flow rate of 0.6 mL/min. The temperature of the column was set at 70 °C, and the RID temperature was set at 35 °C. Elution time was approximately 20 min. 

Data acquisition and the interpretation of results were done by OpenLab—ChemStation (Agilent Techologies, Santa Clara, CA, USA).

The identification of the analysed compounds was carried out by the comparison of retention times with those of standard solutions. Standard solutions (99% purity) of glucose, fructose, and lactic and acetic acid were procured from Merck, Germany.

### 2.6. Flavour Compounds Analysis

Flavour compound analysis was carried out on fresh and fermented chili paste samples. The extraction of the volatile compounds was carried out according to the literature [46] using the ITEX technique. Five grams of each chili sample was introduced in a 20 mL headspace vial. The incubation of the sealed vial was performed according to strict parameters: 60 °C for 20 min under continuous agitation. Volatile compounds from within the vial were repeatedly absorbed (30 strokes) into the porous polymer fiber microtrap (ITEX-2TRAPTXTA, Tenax TA 80/100 mesh). Thermal desorption was performed directly into the GC-MS injector. The hot trap (250 °C) was cleaned with N_2_. The procedures were performed automatically by the CombiPAL AOC-5000 autosampler (Zwingen, Switzerland). All samples were analyzed in triplicate.

The GC–MS analyses were carried out on a GCMSQP-2010 (Shimadzu Scientific Instruments, Kyoto, Japan) model gas chromatograph and mass spectrometer equipped with a CombiPAL AOC-5000 autosampler. The volatiles were separated on a Zebron ZB-5 ms capillary column of 30 m × 0.25 mm i.d. and 0.25 μm film thickness. The carrier gas was helium at 1 mL/min with a split ratio of 5:1 and an injector temperature of 250 °C. The temperature program used for the column oven was: 35 °C (hold for 10 min) to 50 °C at 3 °C/min to 150 °C at 6 °C/min to 200 °C at 10 °C/min and hold for 5 min. The ion-source temperature and interface temperature were set at 250 °C and the MS mode was the electron ionization (EI). The mass range interval scanned was 35–350 μm.

### 2.7. Microbial Assay for Determining the Optimum Moment for the Freeze-Drying of Fermented Chili Paste

Microbial assay was carried out according to recent literature studies [47]. In order to evaluate the dynamics of microbial growth during fermentation, five batches of *C. annuum* cv. Cayenne cultivar chilies were separately fermented for 3, 6, 9, 12, and 15 days. *Cayenne* cultivar chilies were selected based on high carotenoids, vitamin C content, and high pungency in order to be freeze-dried and used in salami processing.

A total of 5 g was collected from homogeneous batches of *C. annuum* cv. Cayenne chili powder. Measured samples were homogenized (Heidoph Reax) in 45 mL of sterile physiological serum. Serial dilutions were carried out (10-fold) in triplicate. An amount of 1 mL of the appropriate dilutions was spread on different (selective and non-selective) agar plates. Microbial assays were carried out for total viable count, LAB, yeasts and molds, and *Enterobacteria*. 

The viability of LAB was determined using MRS (de Man, Rogosa, Sharpe) agar. Total viable counts were analyzed using PCA (Plate Count Agar). Yeasts and molds were grown on DRBC (Dichloran Rose-Bengal Chloramphenicol) agar. Temperatures during incubation (Innova 44) were set as follows: yeasts and molds (30 °C), LAB (37 °C), and total viable counts (30 °C) for a period of a minimum of 48 h. 

Enterobacteriaceae were counted using VRBG (Violet Red Bile Glucose) agar. The incubation temperature was set to 37 °C for a minimum of 48 h. During quantification, only purple colonies were considered. Purple colonies were tested for oxidase activity. Rapid tests (strips) were soaked in deionized water. Purple colonies (pure bacteria) were aseptically transferred on the strip. Color changes from purple to dark blue or almost black indicated positive results. The unchanged color indicated negative results.

I. Determinations for cured salami

### 2.8. Salami Processing

Freshly processed lean pork shoulder and back fat were acquired from a local abattoir and kept in refrigeration conditions (<2 °C) until and during preparation. The weighed materials (meat and fat) were ground through an 8.0 mm plate (DMS; DTX 98), with fat content being adjusted to 30%. The batter was divided into four batches according to the different sources of fermenting microorganisms (Table 1): negative control (B1; spontaneous fermentation); B2-starter culture (Biotec Starter LK 30; Frutarom, Austria); B3-lyophilized fermented chili powder; and B4-fermented chili-paste. Nitrite curing salt was adjusted to an initial value of 2% with a final concentration of 2,8% in the final product (after 28 days). The samples formulation is shown in Table 1, the ingredients were expressed as a 100% mix of ground pork meat (80%) and back fat (20%). The ingredients were purchased from local stores. 

Natural beef casings with a calibrated diameter (Φ = 50–55 mm) were used during preparation (Darimex Techno, Otopeni, Romania). Casings were soaked in lukewarm water, drained, and tied up at one end. The sausage lots were mixed with the spice mixes (Diamond, MCR-30T/N) and stuffed in the casings by a hydraulic stuffing machine (DMS, DF 250 X). The opposite ends were also tied with a string, salami sticks being visually calibrated to approximately 550–600 g. The four batches of salami were each marked and transferred to the climate chamber (Mauting, UKM Junior). Temperature and humidity were strictly controlled, being set according to values presented in the table below. Salami was smoked after 48 h of fermentation and afterward kept in a climate chamber for 28 days (temperature and humidity were strictly controlled).

### 2.9. pH Analysis

The pH of ground meat and salami during maturation was determined using a portable pH meter (Testo 205, Lenzkirch, Germany). This apparatus is adapted to semi-solid products, which also require the automatic adjustment of the reading to different temperatures of the medium. The pH was measured over 28 days, throughout the maturation process of the product. The calibration of the pH meter was performed prior to usage with buffer solutions (pH = 7; pH = 4).

### 2.10. Microbiological Analysis of Salami Samples

A total of 5 g of each cured salami sample (B1–B4) was collected from the core of each stick and subjected to microbial analysis. The same methods were used as presented in Section 2.7.

### 2.11. Texture Profile Analysis of Cured Salami

A specific texture profile analysis (TPA) was performed for assessing the different cured salami samples. The Brookfield CT3 texture analyzer coupled with the TA10 probe (cylinder, 12.7 mm diameter, clear acrylic, 5 g, 35 mm length, sharp edge) was used in the experiment. The test parameters were set as follows: trigger load (0.05 N), target deformation (50%), and test speed (1 mm/s). Experiments were conducted at room temperature.

The uniform slices of salami (48 mm diameter/10 mm thickness) were cut and kept in sterile bags until usage. Samples were compressed twice to 50% of their original height under the enumerated parameters. The force-time curves were used to determine hardness, springiness index, cohesiveness, and chewiness.

Hardness represents the maximum force applied during each compression cycle expressed in N. Springiness is described as the height that salami samples recovered between the end of the first application and the beginning of the second one (expressed in mm). Springiness index (dimensionless, values between 0 to 1), describing the recovery properties of the salami samples and was then calculated as the ratio of Springiness (mm) to the Distance at target (mm), a value of 1 indicating a completely elastic material, and a value of 0 indicating a completely viscous material. Cohesiveness represents the ratio between the areas of the second and the first compression curves, being dimensionless. The product of hardness, springiness, and cohesiveness is described as chewiness, expressed in mJ.

### 2.12. Statistical Analysis

The Duncan multiple correlation test was used (SPSS version19; IBM Corp., Armonk, NY, USA) for the statistical interpretation of scientific data. Fermented and freeze-dried chili samples that showed statistically significant differences were marked with lowercase letters. The results of three independent (*n* = 3) repetitions were expressed as means ± standard deviations. 

## 3. Results and Discussion

I. Results regarding fermented and freeze-dried chili powders

### 3.1. Carotenoids

The carotenoid analysis of fermented freeze-dried chili powder samples showed high concentrations of different molecules mainly in esterified forms with palmitic, lauric, and myristic fatty acids, as presented in Table 2. Statistically significant differences were observed among the examined chili powder samples (*p* < 0.01).

As presented by other studies, capsanthin was found to be the predominant molecule in the examined samples. *C. annuum* cv. Cherry, *C. annuum* cv. Cayenne, and *C. chinense* cv. *Habanero* chilis were shown to contain the highest amounts of carotenoid compounds.

Both free and esterified forms counted for approximately 43–49% of the total carotenoids, except the yellow chili powder (*C. chinense* cv. Fatalii), in the case of which capsanthin represented only 11% of the total. β-carotene represented 34–39% of total carotenoids found in red chili powder samples and 78% in yellow samples (*C. chinense* cv. Fatalii yellow).

Some plants lack the enzyme necessary for the transformation of yellow pigments in red carotenoids [48,49]. This leads to the accumulation of capsanthin precursors (mainly β-carotene) [48], as can be seen in *C. chinense* cv. Fatalii (yellow) samples. β-carotene is also more susceptible to oxidation compared to capsanthin, which leads to higher losses through fermentation [45,50], thus indicating yellow chilis/peppers to be less suitable for chili powder production.

Scientific observations indicate a reduction of carotenoid compounds (from 796.6 mg/100 g to 627.5 mg/100 g) in chili/paprika powder samples if seeds are included in the product [51]. This might explain the slightly lower values in the present study, as whole fruits were used in the experiment.

It has been demonstrated that the initial quality of ground paprika powder is critically important to producing high-quality meat products (e.g., fermented sausages). It was also shown that sun-dried peppers (paprika) provided better stability to the color of Iberian chorizo than the oven-dried spice, also protecting it against lipid oxidation. These variations can be linked to carotenoid degradation during thermal processing (drying) [52]. 

Peppers represent a rich source of carotenoids which are associated with many beneficial properties: protection against chronic diseases, neurodegenerative diseases, free radical scavenging, etc. [53]. Carotenoids within chili or paprika powder also present an important role in meat processing, as they are being used as natural coloring agents that enrich the color of the products (sausages, salami, etc.), known also to exert a protective effect against lipid oxidation [54].

Some carotenoids are exclusively found in pepper fruits (ex. capsanthin, capsorubin, cryptocapsin, etc.) [48,49,55]. Capsanthin was shown to interact with reactive oxygen species, such as superoxide anion radical (O_2_^−^), hydroxyl radical (OH), and singlet oxygen (^1^O_2_), while capsorubin exerted higher stability against these reactive oxygen species [56]. The antioxidant activity of capsanthin might be in direct correlation with the preserving effect of chili powder in meat products, as mentioned above. 

The results of some research articles suggest that capsanthin may become predominant in the final stages of ripening and may acccount for ~60% of total carotenoids [57], thus being considered one of the predominant color components, specific to red pepper fruits (including chili powders). Most carotenoids are shown to be partially or esterified with fatty acids, which increases their molecular stability without interfering with their chromophore properties [48]. This aspect is also confirmed by the present study.

Our findings suggest that fermented chilies resemble the chromophore characteristics of the raw material (fresh chilies) with carotenoids being well preserved during processing. This issue can be correlated with the low drying temperatures, which prevent the degradation of heat-sensitive molecules, as confirmed in other studies [58,59].

However, yellow chilis might require a lower fermenting period, as the stability of β-carotene (specific to yellow chilies) is much lower compared to red pigments (capsanthin).

### 3.2. Phenolic Compounds

An increasing concentration of phenolic compounds in freeze-dried (fermented) chili powder is evident, as shown in Table 3. Registered values ranged between 0.30–0.77%. Freeze-drying showed a good preserving effect on the compounds found in the fermented paste, leading to an increase of the studied compounds in the final product by up to nine-fold [45]. Variations were significant amongst the examined samples. Correlations between phenolics content and pungency were not observed.

A high content of phenolic compounds is desired in chili powder as many of these molecules possess antioxidant [60], antimicrobial [61], and even anti-mutagenic [62] activity. High phenolic content is usually associated with the early stages of ripening [63], fully matured fruits showing slightly reduced concentrations compared to the premature (unripen) form of the fruit [45,62].

Variation of bio-compounds is also influenced by the inclusion or absence of the different partitions of the fruits in the experiment. The inclusion of seeds in the chili powder reduces total phenolic content [64] but has a preserving effect on color compounds due to the presence of tocopherols [51]. Tocopherols are lipophilic molecules concentrated in the oil-rich parts of the seeds [65]. 

Many studies discuss the importance of phenolic compounds being incorporated into food products. The diversity and complexity of phenolic compounds assures a wide spectrum of benefits for the products and the consumer as well. Numerous studies have evaluated the antimicrobial activity of different phenolic-rich extracts against pathogens [20,66,67]. However, some studies underline the fact that, besides the inhibition of pathogens, phenolic compounds can also inhibit beneficial (starters) bacteria growth in fermented products (*Staphylococcus xylosus*; *L. curvatus*, *Pediococcus pentosaceus*) [68] if used in high concentration.

According to an experiment, regarding phenolic compounds in fermented meat products, the incorporation of different phenolic extracts (75–150 mg/100 g dough) in ground meat exerted a clear antioxidant effect in the food matrix (salami) [69]. Another study indicated that during the aging process approximately 54–61% of the initial catechin and epicatechin content were lost in dried fermented sausages [70]. Losses amongst different molecules varied greatly.

In this regard, the preserving of phenolic compounds under the effect of freeze-drying techniques in fermented chili powders is extremely important due to the role of these molecules in later stages, when incorporated in different products (food matrices).

### 3.3. Ascorbic Acid

The present study evaluated the ascorbic acid content of lyophilized chili powder obtained from fermented chili paste that was spontaneously fermented for 21 days. Three weeks were counted as the maximum that were considered. The optimum fermentation period is also conditioned by the microbial composition and dynamics of the microbial community. In this regard, the total losses of vitamin C content through the fermenting process were quite low (~20%), as was recorded in our previous study [45]. 

The ascorbic acid content in the chili powder samples was high, losses being prevented primarily by the drying method (freeze-drying). Average concentrations were within the limits of 0.40–1.11%, as presented in Table 4:

As confirmed by the present results, *C. annuum* cv. Cayenne peppers tend to contain the highest amount of ascorbic acid. This pattern is also highlighted by other studies [71].

Results confirm the advantages of the freeze-drying technique as being one of the best for preserving valuable compounds in dehydrated products [72] and also show the applicability in capsicum processing.

According to the literature, vitamin C content is strongly susceptible to negative changes during thermal stress. Some experimental data showed a powerful decrease in ascorbic acid from 911 ± 50.1 mg/100 g (drying temperature = 50 °C) to 87.9 ± 2.8 mg/100 g (drying temperature = 90 °C) due to temperature variations [15]. It is also known that variations in the vitamin C content of ground paprika can vary according to processing techniques and preservation conditions [4]. 

Differences amongst samples can occur naturally due to endogen and exogen factors but variations between partitions of the fruits can also have a dominant effect. Generally, whole-ground fruits contain less ascorbic acid than powders obtained from dried pulp [73].

Ascorbic acid is generally used in meat processing as an additive under different forms: E300 (ascorbic acid), E301 (sodium ascorbate), E302 (calcium ascorbate), E303 (potassium ascorbate), and E304 (fatty acid esters of ascorbic acid). Its primary role is to prevent the oxidation and discoloration of the product and to delay the appearance of the unpleasant color [74]. Some studies also discuss the nitrite scavenger activity of ascorbic acid with possible implications in the prevention of nitrosamine formation [75]. As a chemical reducing agent, ascorbic acid can reduce nitrites and accelerate the formation of meat pigments (nitroso myoglobin), thus increasing the antibacterial activity of nitrite [76].

The addition of fermented chili powder rich in ascorbic acid might serve as a stabilizing agent in fermented meat products, thus excluding the necessity of supplementation from external sources.

### 3.4. Sugars and Acids

Fermented chilies showed increased acid content, especially lactic acid. In a previous article, we discussed these issues [45]. The lactic acid content of different types of chilies ranged between 1.03–1.31% (lactic acid) and acetic acid was present in the limits of 0.32–0.49% [45]. The lyophilization of the paste increased lactic acid concentration by up to 10 folds. Fermented chili powder showed high lactic (10.57–12.20%) and acetic acid concentrations (3.39–4.10%), as shown in Table 5.

The glucose content of fermented and dehydrated chilies was very low in all samples, ranging in the limits of 0.28–2.27%. These results might indicate an affinity of plant-specific *Lactobacillus* sp. towards glucose as the primary carbohydrate source. In a previous study, we presented fructose as to be found in similar concentrations both in fresh and fermented peppers [45]. Contrary to glucose, fructose was only partially metabolized in the fermenting process [77], which led to the concentration of the compound in chili powder (12.59–19.30%). 

Acidic compounds showed an inverse trend. Both lactic and acetic acid were absent or found in minimal concentrations in the fresh samples. Fermentation and dehydration had a concentrating effect on the final product, as lactic acid reached concentrations of 10.57–12.20%. Acetic acid also increased up to 3.39–4.10%. These aspects are important, as naturally occurring acidic compounds incorporated in food products by the addition of chili powder might speed up the acidification process, which could have a shortening effect on the time necessary for the stabilization of the product. Fructose might also serve as a carbon source for encapsulated microorganisms during the adaptation period and the log phase.

A study conducted on different paprika powder samples showed variable results among them regarding carbohydrate content. The average glucose content was 21.5%, whereas fructose content was slightly higher (31.6%) [10]. These results are quite different compared to the results of our experiment (fermented chili powder), indicating significant transformations in the biochemical composition of the matrix. 

The presence of simple carbohydrates is essential for the fermenting activity of LAB. The acid formation is crucial for the preservation of many products, including dairy and fermented meat. Weak acids can penetrate the hydrophobic membrane of pathogens and dissociate in the cytoplasm as their concentration increases, thus showing a burden on cell metabolism by the reduction of plasma pH [35]. Salami fermentation implies similar patterns. Spontaneous or selected microorganisms (starter cultures) that are adapted or adaptable to protein substrates, multiplicate and colonize the stuffing. Sugar metabolism and acid synthesis lead to the drop of pH and stabilization of the produce. Humidity loss during maturation decreases water activity, further inhibiting pathogens [42,78]. 

In this regard, carboxylic compounds formed during sugar metabolism in chili paste might play a critical role in fermented meat products, especially during the lag phase (first 24–48 h of incubation), when the substrate is the most susceptible to contamination.

### 3.5. Aroma Profile

Volatile fingerprinting was conducted to identify compounds specific to aroma formation involving a two-step interpretation. First, *C. annuum* and *C. chinense* species were compared in the fresh and fermented stages to confirm common trends or differences between the two. Secondly, chili samples were compared individually in both unfermented and fermented forms. In the last stage, individual samples were analyzed and compared to identify aroma profile development threw fermentation.

**Figure 1:**
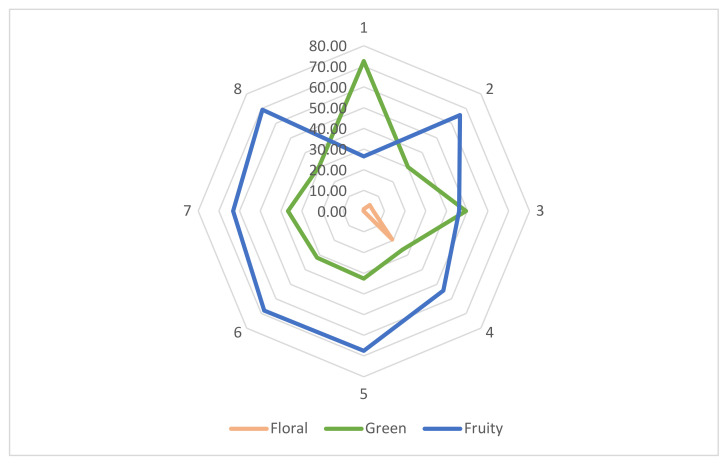
Sensory mapping of *Capsicum* specific aroma compounds: 1 (cv. Cherry/fresh); 2 (cv. Cherry/fermented); 3 (cv. Cayenne/fresh); 4 (cv. Cayenne/fermented); 5 (cv. Fatalii/fresh); 6 (cv. Fatalii/fermented); 7 (cv. Habanero/fresh); 8 (cv. Habanero/fermented).

A total of 130 different volatile compounds (as shown in the Table S1, Supplementary Materials section) were identified in the examined samples (shown in Figure 1) with many esters, which according to the literature are responsible for the fruity notes in aroma development [79]. *C. chinense* samples were shown to be more complex regarding volatile compounds than *C. annuum*. Studies underline this fact and some even show significant differences among different cultivars within the same species. One study indicates that orange and brown cultivars of *C. chinense* cv. Habanero chilies contain a higher amount of volatiles (esters) than red cultivars [80]. A general view regarding the origin of volatile compounds does not yet exist. However, studies show for example that 2-methylbutanoic acid and 2-methylpropionic acid are mainly derived from branched chained amino acids, including valine, leucine, and isoleucine [81]. These processes involve the metabolic activity of different microorganisms and require different reactions (transamination, oxidation, etc.) [82]. 

Results show differences amongst species (*p* < 0.05), but a common pattern is observed amongst peppers related to the same species. *C. chinense* presents a more complex volatile profile, being characterized by a fruity/exotic, citrus-like aroma [79]. The more complex profile is also evident in all fermented samples in contrast to the fresh chilies.

Total compounds identified in unfermented and fermented *C. annuum* cultivars are not significantly different (*p* < 0.05). Fresh *C. annuum* cv. Cherry type chilies present 25 volatile compounds with an increase to 28, whereas compounds in *C. annuum* cv. Cayenne type peppers increase from 18 to 25. However, a great part of compounds identified in the first stage may not appear in the fermented form, thus being substituted with new ones.

The major volatiles identified in *C. annuum* pepper samples were hexane derivatives, 2-hexenal, E-2-hexen-1-ol, and 1-hexanol. These compounds presented a universal and relatively uniform distribution amongst *C. annuum* cultivars. 2-hexenal is predominant, and according to the literature, it is the main aldehyde responsible for the sweet fruity aroma of chilies. Pepper fermentation in the case of *C. annuum* cv. Cherry and *C. annuum* cv. Cayenne chilies did not significantly (*p* < 0.05) affect the two compounds 1-hexanol and E-2-hexen-1-ol. In fact, in the case of *C. annuum* cv. Cherry type peppers there was an increase in E-2-hexen-1-ol from 12.98 ± 0.65% to 26.06 ± 1.30%. Fermentation also leads to the acetylation of E-2-hexen-1-ol with the formation of E-2-Hexen-1-ol acetate, specific exclusively to fermented samples. However, 2-hexenal, the predominant compound could not be identified in the fermented samples of the mentioned cultivars. These results likely indicate that the only the alcohols and aldehydes of sensory importance found in *C. annuum* species were 1-hexanol, 2-hexanol, hexenal, and E-2-hexenal. 

Linalool was only identified in fermented *C. annuum* samples. This terpene alcohol is usually associated with a floral scent/citrus-like aroma, combined with mild spiciness. The molecule is normally synthesized from geranyl pyrophosphate by the enzyme linalool synthase. Studies indicate that linalool represents a key aroma-active compound in pickled red peppers [19] amongst others (acetic acid, ethanol, a-terpineol, (E)-2-nonenal, 2-heptanol, phenylethyl alcohol). Amongst newly formed compounds, n-propyl acetate was the more predominant. This ester was only identified in fermented *C. annuum* samples and was more predominant in *C. annuum* cv. Cayenne type peppers (9.11 ± 0.46%). The literature describes propyl acetate odor as mild, pleasant, and fruity (pear-like).

Alcohols in general present a higher odor threshold in comparison with other molecules (aldehydes, acids). This is the reason why their role in aroma formation is much lower [18]. Amongst the aldehydes identified in this study that might account for the development of aroma of pepper fruits, there are a few compounds to be mentioned: hexanal, pentanal, benzaldehyde, etc. The first two are described as green, pungent, and herbaceous odors [18]. 

Benzaldehyde is a compound universally distributed amongst all samples (fresh/fermented) in both species in various concentrations (0.01–2.72%), which is known to add an almond type odor. Acetophenone was also identified in all samples (0.03–4.28%). This volatile compound stands for a sweet/pungent orange-like odor of different fruits and vegetables.

*C. chinense* includes cultivars with a broad spectrum of volatile compounds that overlap the composition of *C. annuum* cultivars. Differences are statistically significant (*p* < 0.05), both between fresh samples and fermented samples. As evidenced by the results, *C. annuum* can more likely be correlated with alcohols as primary volatile compounds, whereas *C. chinense* is represented by organic acids. Aldehydes and acids present a lower sensory threshold, thus contributing to the more predominant aroma development of *C. chinense* fruits and fermented chili samples in general.

*C. chinense* cv. Fatalii chilies contain 49 volatile compounds, which, due to fermentation increase to 60. *C. chinense* cv. *Habanero*, has 34 compounds in the fresh stage and reaches 48 after fermentation. The literature indicates that *C. chinense* cv. Habanero red-type peppers (like our samples) show a lower aromatic profile in comparison with orange or brown types, similar to the data shown by our research.

The predominant volatile compounds specific to *C. chinense* samples were butanoic acid, 3-methyl-, and hexyl ester, in both fresh and fermented pepper mash. This molecules presents a tropical, fruity, long-lasting fragrance. Butanoic acid, 2-methyl-, and hexyl ester were also present in all samples in quite similar concentrations (9.08–12.3%), having similar tropical, fruity aroma characteristics. Interestingly, butanoic acid and 2-methyl- hexyl ester were also identified in all fresh and fermented *C annuum* samples in smaller concentrations (0.69–5.24%) and are thus considered to be a generally occurring *Capsicum*-specific volatile compound, which is more predominant in *C. chinense* species, thus contributing to its tropical, citrus-like fresh fruity flavor.

Amongst alcoholic compounds, 1-pentanol,4 methyl- is the more predominant form, ranging between values of 2.64–6.06%. This molecule was also found to be evident in all *C. annuum* samples (both fresh and fermented) in a range of 1.11–7.36%.

### 3.6. Determining the Optimum Moment for Freeze-Drying of Fermented Chili Paste

The controlled fermentation of chili peppers is quite difficult, as starter cultures are less efficient and cannot compete with spontaneous flora, being rapidly outnumbered and inhibited, especially if the process is carried out at higher temperatures [83]. Some studies have reported the use of autochthonous strains as starter cultures with quite good results [84]. However, pasteurization or any tendency to sterilize the medium equals with the loss of significant amounts of bio-compounds (ascorbic acid, carotenoids, phenolic compounds, etc.) [15,51], whereas the endogenous microflora of vegetables is usually well adapted and can outgrow starter cultures quite rapidly [85]. Microbiologically, vegetable fermentation implies many phases in between which different changes occur in the microbial community. 

We presumed that fermented pepper mash should be lyophilized in the first phase of fermentation in order to concentrate high amounts of bio-compounds and thus to obtain high levels of organic acids and flavor compounds, yet to prevent yeast growth, which is not necessarily desired in fermented meat, although yeast loads in fermented sausages can sometimes reach 10^6^ UFC/g [27,86]. The dynamics of the microbial community during a 15-day period, conducted on *C. annuum* cv. Cayenne peppers are described in Table 6. *C. annuum* cv. Cayenne cultivar chilies were selected based on high carotenoids, vitamin C contents, and high pungency to be freeze-dried and used in salami processing.

Changes are quite evident as described above. Results for total viable counts are close to values obtained for LAB. This means that after fermentation is initiated in chilies, LAB becomes the predominant microorganisms in the substrate, as their adaptability far outreaches the growth of other species. Our previous research describes the propagation phase in chili fermenting processes, which highlights the importance [45] of spontaneous microbial flora. The initial values (lag phase) for bacterial counts in fresh pepper paste is usually <10^3^ CFU/g [45]. Microbial growth (log phase) initiation takes place in a maximum of 24–48 h and is only partially conditioned by the capsaicinoid content of pepper fruits. In this regard, highly pungent chilies may prolong the lag phase up to 48–72 h. However, as it is presented below, the results regarding the viability of LAB in fermented peppers, including the lyophilized version, are quite similar. The decreasing numbers after lyophilization are evident, but moderate, viable cell concentrations still outreach 10^7^ CFU/g in pungent and highly pungent samples. 

The comparison of the previous results indicates a very stable stationary phase of LAB in fermented peppers, with a continual concentration of yeasts during the process.

According to the presented results, we consider that days 4–6 would be optimum (from a microbiological perspective) for the freeze-drying of the fermented paste to obtain a highly concentrated matrix suitable for salami production.

**Figure 2:**
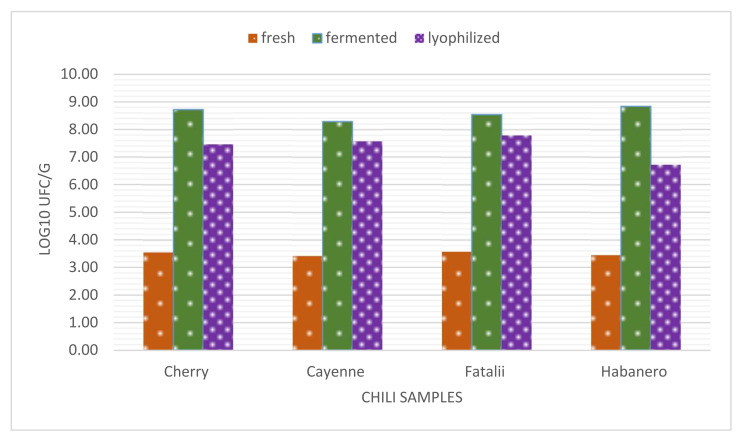
Diagram representing viability of LAB in peppers of different pungencies in different stages (fresh; fermented 21 days and freeze-dried). In this case, spontaneous fermentation was carried out for a maximum of 21 days.

Figure 2 also highlights the changes in LAB dynamics in different stages of fermented chili powder production. Final counts in the dried powder are only slightly lower than those counted for fermented chili paste. This underlines the importance of parameter control during freeze-drying in order to prevent bacterial cell damage.

LAB showed a tendency towards concentration during the process that persisted throughout the 15-day period. Yeast counts presented very low values in the beginning. Although their number increased exponentially, their yeast cells were permanently outnumbered by bacteria during the experiments. Other studies indicate similar patterns regarding microbial activity in vegetable fermentation. It is commonly accepted that the initiation of the process is carried out by *Leuconostoc* species [87], which leads to the rapid decrease of pH values [7]. The new conditions (anaerobiosis) favor the growth of *L. plantarum* [88] and the development of other microorganisms (yeasts, *Pediococcus cerevisiae*, *L. brevis*, *Weisella*, etc.). 

In this regard, we concluded, that the freeze-drying of fermented chilies might be carried out in the first 6 days. This period coincides with total carbohydrate metabolism and maximum reach in organic acid concentration combined with a relatively low yeast concentration.

It is also clear that *Enterobacteriaceae* show no changes as their activity is suppressed by LAB activity. The contamination of pepper mash or chili powder, in this regard, might be primarily conditioned by the quality of raw material and applications of good hygiene practices.

I. Results regarding cured salami

### 3.7. pH Analysis in Salami Fermentation

Fermented meat products (sausages, salami, etc.) require a rapid increase of acidity to stabilize the product and to prevent the development of spoilage and pathogen microflora. It is confirmed that initial pH, usually >5.5, stagnates at approximately 48–72 h (lag phase) [22], afterwards, under intense microbial activity (log phase) and pH values drop steeply (pH < 4.6–4.8). The last phase is characterized by the stabilization of pH (4.8–5.00) and partial neutralization of organic acids by free amino acids (the result of protein hydrolysis) that can act as buffering agents. Final a_w_ values decrease to approximately 0.88, leading to increased stability and long shelf life [31]. Studies indicate that the microbiota of fermented meat products usually consists of LAB *(L. sakei*, *L. curvatus*, and *L. plantarum*) and coagulase negative cocci (ex. *Staphylococcus xylosus*) [22,28].

The pH variations of fresh meat and different batches of the batter (prior to stuffing) are presented in Table 7.

**Figure 3:**
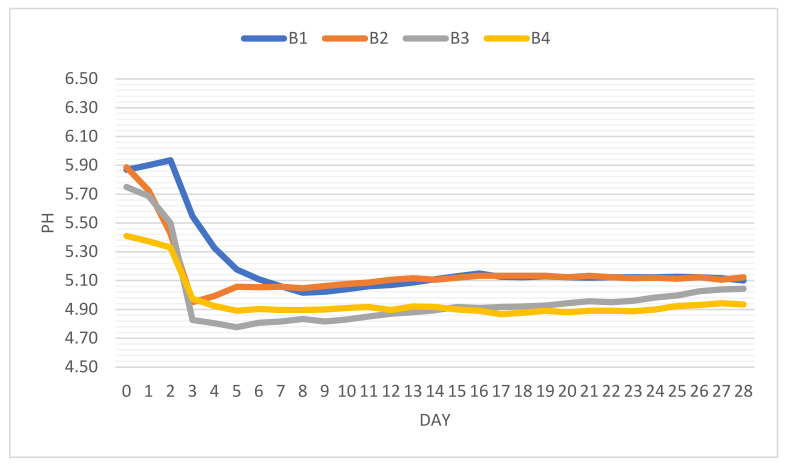
Representation of pH variation during the fermentation and drying process of four batches of salami, obtained with different sources of LAB.

Fermented chili powder acts as a strong acidifier decreasing the pH values of the batter due to high organic acid content (lactic and acetic acid), as is evident in case of sample B3. H^+^ ions were the most active in B4, in the case of which pH decreased by approximately 0.77 basis points. Fermentation (the first phase of conditioning) is usually carried out (18–20 °C, 90% relative humidity) until the pH drops under 5.2. In this regard, the incorporation of fermented vegetable powders (chili) can act as natural acidifiers that can also enhance LAB activity. The variation of pH during the 28-day processing period is depicted in Figure 3. 

In the case of B1 (negative control), the pH drop relies on low concentrations of endogenous microflora, which leads to the expansion of the lag phase with values <5.2 being reached only after 5 days. This time interval would have been critical, especially if the microbiological quality of the raw material would have been poor. The other three batches showed remarkedly similar patterns, as their pH drop was almost spontaneous and steep. In less than 72 h the pH dropped to under 5.00. Fermented freeze-dried chili powder led to values of 4.83 in the case of B3. 

Fermented chili paste increased the water content of the stuffing with humidity values reaching up to 63% in comparison with the average content in the other three cases (57%). This sample (B4) also presented the best dynamics in pH variation. It is not a common practice to use pepper paste in salami production, although it is not excluded. Many Italian-type sausage or salami recipes imply the usage of wine as an ingredient.

From the moment that minimum pH values were reached, an appreciating trend was observed with final values (after 28 days) being registered as follows: 5.10 (B1); 5.12 (B2); 5.04 (B3); 4.93 (B4).

### 3.8. Microbial Analysis

Results indicate correlations between LAB growth and pH variations. The first phase of conditioning (incubation period) had a huge impact on the outcome of the process, as it involved bacterial growth under physical conditions that can favor pathogens (t = 18–20 °C; h = 90%). 

Different cell loads were determined prior to stuffing: 5 × 10^3^ CFU/g (B1); 2.7 × 10^5^ CFU/g (B2); 1.14 × 10^6^ CFU/g (B3); and 4.2 × 10^7^ CFU/g (B4). These data confirm the low numbers of LAB in the raw material (meat), as can be seen in B1 (negative control). At this stage, LAB was highly outnumbered by endogenic microbiota in (B1). 

**Figure 4:**
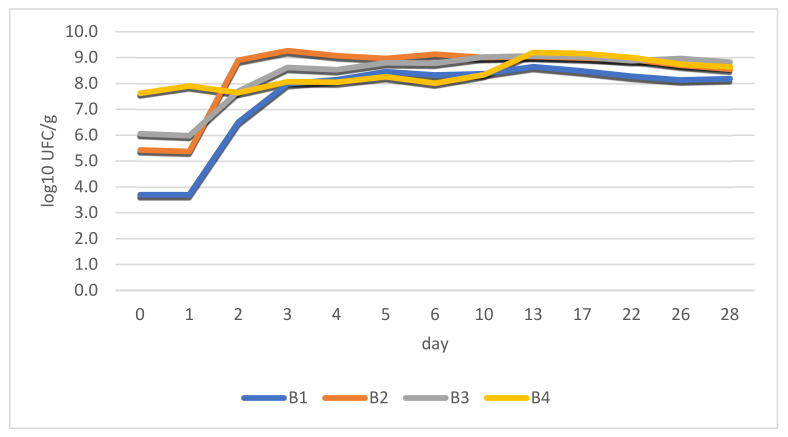
LAB growth during the 28-day fermentation period in the case of four batches of fermented salami with different sources of fermenting bacteria.

It was noticed that the lag phase lasted for approximately 24 h afterwards and exponential cell growth was observed for 48 h. The stationary phase (maximum load) was reached in all cases after 72 h_._ There was an exception, however, regarding the B4 sample. In the case of this batch, fermented paste replaced dried chili powder, which delivered a relatively constant bacterial load right from the beginning. This aspect is revealed in Figure 4.

The stability of the products was observed during the stationary phase throughout the entire period (28 days) with results being quite close to each other. 

A similar experiment was carried out, which focused on the incorporation of fermented vegetables in meat products [42]. The authors of this experiment partially reduced curing salt concentration to prevent bacterial growth inhibition. Our results indicate that bacteria from all sources presented excellent halotolerance, even towards the final stages, when NaCl concentrations reached 2.8%. In most cases, the spontaneous fermentation of meat products is carried out by microorganisms that are to be found in different plant sources [22], including fermented vegetables. The spontaneous fermentation of salami can also be influenced by bacteria preserved in highly saline conditions found on natural casings [27].

**Figure 5:**
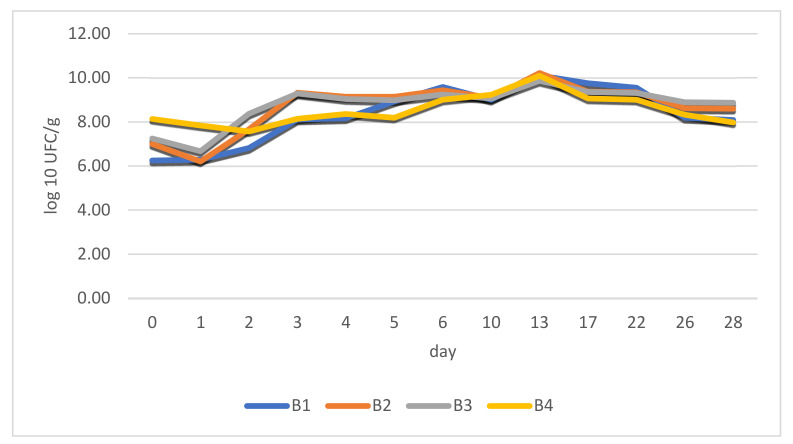
Total viable count during a 28-day fermentation period in the case of four batches of fermented salami with different sources of fermenting bacteria.

Figure 5 identifies changes in total viable counts. Colony counting showed cell loads of approximately 1.79 × 10^6^ CFU/g for B1. This confirms a low concentration of LAB in the ground meat at this stage (B1) and high numbers of endogenous microbes.

Total counts reached 1.0 × 10^7^ CFU/g (B2), 1.75 × 10^7^ CFU/g (B3), and 1.36 × 10^8^ CFU/g (B4) after the addition of spices (before stuffing). At this stage LAB from different sources outnumbered the original microbial population by 10:1 (at least), as expected [31,35].

An increasing pattern for values is observed as LAB concentrate in different sources (starter culture, fermented chili, and fermented chili paste), becoming the predominant microorganism in the salami. The high concentrations of fermenting bacteria are needed from the start of the process in order to numerically outgrow the existing microflora and prevent spoilage.

As the fermentation process carries on, numerical data from total viable counts and LAB begin to overlap each other. This means that in this stage LAB becomes dominant and represents most of the counted microorganisms.

Yeast counts and *Enterobacteriaceae* both presented constant values (<10^2^ CFU/g) during the ripening period. The presence of some yeast species and their numeric variation in meat products is confirmed [42], although counts were very low in this experiment. They usually consist of different species, such as *Debaryomyces hansenii*, *Candida zeylanoides*, *Pichia triangularis*, etc. [22,89]. The reduced values in the case of yeast counts in salami samples might be associated with low values in fermented chili samples just before lyophilization (chili powder production). The extended fermentation of chilies might have led to higher concentrations of yeast cells in chili powder and salami as well.

*Enterobacteria* are usually associated with contaminated meat, whereas meat spoilage requires the overcoming of competitiveness of pathogens by high loads of beneficial bacteria (starters) [2,42]. *Enterobacteria*, although present in cured products in different concentrations, their growth is usually inhibited in the first few days as a consequence of the metabolic activity of LAB [28].

### 3.9. Texture Profile Analysis

Acidification plays an important role in the formation of color, whereas protein coagulation increases firmness and cohesiveness [90]. Immediately after stuffing, the product shows low cohesiveness, as it has a crumbly texture. Lactic acid synthesis carried out by different LAB leads to the drop of pH values to a point close to the isoelectric values of muscle protein. Consequently, moisture retaining capacity drops, favoring the dehydration of the produce.

Salami-like (semisolid) texture comes as a result of humidity loss and protein (sarcoplasmic and myofibrillar) coagulation [91]. 

Results for TPA analysis showed statistically significant variations amongst the analyzed samples (*p* < 0.05) as presented in Table 8. Although the curing process was carried out in similar conditions, differences did occur. These variations might be correlated with different factors.

Normal chili/paprika powder has a humidity of approximately 8–11% [92], lyophilized fermented chili powder was extremely dry, whereas fermented chili paste has a humidity of approximately 90%. Bacterial interactions (bacteria/substrate), carbohydrate content, ripening conditions, and other factors can also influence the texture profile of the end product [32,90].

The lowest values for cohesiveness were found in salami fermented with chili paste (B4). This might indicate the necessity of longer maturation periods to facilitate slow and constant evaporation of excess moisture.

The use of the starter culture increased the hardness of the salami, showing maximum values for all determinations. 

The correlation of powders with their humidity values might be taken into consideration to appropriately assess the exact doses for spices to obtain similar characteristics. Good cohesiveness and hardness also imply the moderate usage of dry powders in fermented meat products as powders tend to destabilize the matrix.

## 4. Conclusions

This research studied the effects and possible applications of freeze-dried fermented chili powders incorporated in cured meat products (fermented meat). Besides the coloring and spicing effect, fermented chili powder shows the ability to immediately and significantly affect the pH of the medium that is added to, serving as a natural acidifying agent. It also serves as a natural starter carrier needed for the initiation of fermenting activity, carried out by LAB in the filling (sausage, salami, etc.). Plant-specific LAB showed great adaptation to the protein substrate (fermented salami), exceptional halotolerance, and proliferation under the new conditions. LAB counts reached >10^9^ CFU in the salami samples, results like those obtained by the usage of technological starter cultures. All bio-compounds were found to be well preserved in freeze-dried fermented chilies, the quality of the spice being correlated with the biochemical composition of fresh chilies, and the length of the fermenting period. Freeze-dried fermented chili powder might serve as an improved spice that can act as a stabilizing agent in fermented meat products, being able to eliminate the necessity of pure bacterial strain usage. It might also improve the aroma profile and resemble the properties of more traditional (hand crafted) products. Future studies will be conducted for more in-depth determination regarding the influence of freeze-dried fermented chili powder on meat products and with an increased number of replications. 

## Figures and Tables

**Table 1:** Formulations of salami samples and their codifications according to the different sources of fermenting microorganisms used during processing.

Ingredients (%)	B1	B2	B3	B4
Pork shoulder	80	80	80	80
Back fat	20	20	20	20
Curing salt	2	2	2	2
Chili powder (traditional)	2	2	-	-
Freeze-dried (fermented) chili powder	-	-	2.0	-
Fermented chili paste	-	-	-	10
Biotec Starter LK 30	-	0.00025	-	-
Dextrose	0.5	0.5	0.5	0.5
Black pepper	0.4	0.4	0.4	0.4
Garlic	0.25	0.25	0.25	0.25
Coriander seeds	0.1	0.1	0.1	0.1

**Table 2:** Variation of carotenoid compounds in the samples of freeze-dried fermented chili powder.

Chili Sample/Carotenoid Compounds	*C. annuum* cv. Cherry	*C. annuum* cv. Cayenne	*C. chinense* cv. Fatalii	*C. chinense* cv. Habanero
Capsanthin	46.67 ± 1.05 ^c^	97.80 ± 2.33 ^e^	12.02 ± 0.20 ^a^	29.89 ± 1.03 ^b^
Zeaxanthin	97.72 ± 3.16 ^c^	106.49 ± 3.11 ^e^	24.38 ± 1.02 ^a^	55.23 ± 1.11 ^b^
β Cryptoxanthin	179.42 ± 2.36 ^d^	184.99 ± 4.19 ^e^	17.00 ± 0.07 ^a^	120.68 ± 2.37 ^c^
Capsanthin-myristate	241.09 ± 2.09 ^e^	210.50 ± 5.92 ^c^	3.61 ± 0.01 ^a^	195.35 ± 5.02 ^b^
Capsanthin-palmitate	142.69 ± 0.13 ^c^	143.78 ± 3.13 ^d^	11.13 ± 0.03 ^a^	176.74 ± 0.91 ^e^
β Carotene	880.61 ± 9.33 ^e^	840.74 ± 9.37 ^d^	422.45 ± 4.12 ^a^	836.59 ± 8.07 ^c^
Capsanthin-di-laureate	109.33 ±1.26 ^c^	128.09 ± 4.55 ^d^	6.73 ± 0.04 ^a^	140.16 ± 3.99 ^e^
Capsanthin-laureate-myristate	177.14 ± 2.32 ^d^	201.89 ± 3.81 ^e^	6.28 ± 0.01 ^a^	150.56 ± 4.57 ^c^
Capsanthin-di-myristate	204.31 ± 3.95 ^c^	239.80 ± 5.06 ^d^	14.94 ± 1.02 ^a^	267.61 ± 8.11 ^e^
Capsanthin-myristate-palmitate	93.59 ± 1.07 ^c^	128.78 ± 1.23 ^e^	4.60 ± 0.02 ^a^	96.26 ± 2.03 ^d^
Capsanthin-di-palmitate	50.58 ± 0.33 ^d^	47.86 ± 0.77 ^c^	0.39 ± 0.00 ^a^	52.01 ± 1.11 ^e^
Zeaxanthin-di-myristate	107.89 ± 2.85 ^d^	61.54 ± 1.09 ^c^	8.80 ± 0.12 ^a^	120.85 ± 3.25 ^e^
Zeaxanthin-myristate-palmitate	75.41 ± 0.99 ^e^	42.02 ± 1.01 ^c^	6.42 ± 1.03 ^a^	50.14 ± 0.80 ^d^
Zeaxanthin-di-palmitate	55.80 ±0.48 ^d^	21.60 ± 0.41 ^c^	5.44 ± 0.01 ^a^	15.53 ± 1.27 ^b^
Total carotenoids	2462.22 ± 10.51 ^e^	2455.86 ± 12.51 ^d^	544.18 ± 6.71 ^c^	2307.61 ± 16.01 ^a^

Values (µg/g) represent means ± standard deviation of the mean for three replicates. Note: The different letters indicate a significant difference (*p* < 0.05) between the carotenoids in the same line (horizontal).

**Table 3:** Variation of total phenolic compounds in the samples of fermented freeze-dried chili powder.

Chili Sample	Total Phenolic Content
*C. annuum* cv. Cherry	771.26 ± 12.05 ^b^
*C. annuum* cv. Cayenne	425.28 ± 7.38 ^c^
*C. chinense* cv. Fatalii	306.52 ± 4.99 ^d^
*C. chinense* cv. Habanero	301.84 ± 8.48 ^d^

Values (mg/100 g) represent means ± standard deviation of the mean for three replicates. Different lower-case letters indicate statistically significant differences amongst chili cultivars.

**Table 4:** Variation of ascorbic acid content in the examined freeze-dried fermented chili powder samples.

Chili Sample	Total Ascorbic Acid
*C. annuum* cv. Cherry	398.39 ± 4.17 ^b^
*C. annuum* cv. Cayenne	1107.33 ± 2.31 ^c^
*C. chinense* cv. Fatalii	499.03 ± 4.54 ^d^
*C. chinense* cv. Habanero	585.05 ± 6.09 ^e^

Values (mg/100 g) represent means ± standard deviation of the mean for 3 replicates. Different lower-case letters indicate statistically significant differences amongst chili cultivars.

**Table 5:** Variation of sugar and organic acid content of freeze-dried fermented chili powder samples.

Chili Cultivar	Glucose	Fructose	Lactic Acid	Acetic Acid
*C. annuum* cv. Cherry	2.83 ± 0.60 ^a^	175.77 ± 4.55 ^b^	112.62 ± 4.22 ^a^	40.77 ± 2.55 ^c^
*C. annuum* cv. Cayenne	9.02 ± 0.51 ^c^	193.00 ± 5.33 ^d^	121.96 ± 4.28 ^c^	41.00 ± 2.33 ^c^
*C. chinense* cv. Fatalii	22.31 ± 2.03 ^d^	125.93 ± 9.49 ^e^	105.74 ± 2.03 ^d^	33.93 ± 3.49 ^a^
*C. chinense* cv. Habanero	22.71 ± 1.04 ^d^	159.01 ± 3.58 ^f^	117.58 ± 4.54 ^e^	34.01 ± 3.58 ^a^

Values (mg/g) represent means ± standard deviation of mean for three replicates. Different lower-case letters indicate statistically significant differences amongst chili cultivars.

**Table 6:** Changes in microbial community during chili fermentation (CFU/g) conducted on *C. annuum* cv. Cayenne type chilies at different times.

Microbial Assay	Day 3	Day 6	Day 9	Day 12	Day 15
Total viable count	3.40 × 10^7^	4.11 × 10^7^	7.33 × 10^7^	6.72 × 10^8^	8.70 × 10^8^
LAB	3.30 × 10^7^	4.00 × 10^7^	6.90 × 10^7^	6.60 × 10^8^	8.66 × 10^8^
Yeasts	<10^3^	8.13 × 10^4^	1.03 × 10^5^	3.33 × 10^5^	2.8 × 10^6^
Enterobacteriaceae	<10^2^	<10^2^	<10^2^	<10^2^	<10^2^

Values (UFC/g) represent means for 3 replicates.

**Table 7:** pH variations before/after spicing the minced meat (before stuffing).

	FM	B1	B2	B3	B4
Measured pH	6.18 ± 0.16	5.87 ± 0.06	5.89 ± 0.04	5.75 ± 0.03	5.41 ± 0.02

FM (fresh meat); B1 (first batch/negative control); B2 (second batch/starter culture used); B3 (third batch/fermented chili powder added); B4 (fourth batch/fermented chili sauce added).

**Table 8:** Results of texture profile analysis (TPA) for fermented salami with different sources of fermenting bacteria.

Samples	Hardness Cycle 1 (N)	Hardness Cycle 2 (N)	Cohesiveness	Gumminess (N)	Springiness Index	Chewiness (mJ)
B1	29.30 ± 1.97 ^d^	25.25 ± 1.77 ^b^	0.54 ± 0.03 ^c^	15.65 ± 0.73 ^b^	0.75 ± 0.05 ^a^	71.13 ± 7.45 ^d^
B2	54.43 ± 4.63 ^b^	46.05 ± 3.19 ^c^	0.49 ± 0.05 ^d^	26.65 ± 2.09 ^c^	0.78 ± 0.03 ^b^	113.68 ± 11.26 ^b^
B3	37.35 ± 5.42 ^c^	31.96 ± 4.25 ^d^	0.45 ± 0.07 ^b^	16.46 ± 1.64 ^a^	0.67 ± 0.07 ^c^	57.98 ± 10.11 ^c^
B4	39.89 ± 4.00 ^a^	34.25 ± 2.97 ^a^	0.40 ±0.03 ^a^	15.99 ± 1.32 ^a^	0.69 ± 0.11 ^d^	62.83 ± 10.45 ^a^

Values represent means ± standard deviation of the mean for three replicates. Different lower-case letters indicate statistically significant differences amongst salami samples.

## Data Availability

Data is contained within the article or Supplementary Material.

## Supplementary Materials

The following supporting information can be downloaded at: https://www.mdpi.com/article/10.3390/foods11223716/s1, Table S1. Variation of aroma compounds in the examined samples of fresh and fermented chilies (%) (Values (%) represent means for 3 replicates; n.i.—not identified).

## References

[1] Pawar S., Bharude N., Sonone S., Deshmukh R., Raut A., Umarkar A. (2011). Chillies as food, spice and medicine: A perspective. Int. J. Pharm. Biol. Sci..

[2] Colavita G., Piccirilli M., Iafigliola L., Amadoro C. (2014). Levels of Nitrates and Nitrites in Chili Pepper and Ventricina Salami. Ital. J. Food Saf..

[3] Ikonić P., Jokanović M., Petrović L., Tasić T., Škaljac S., Šojić B., Džinić N., Tomović V., Tomić J., Danilović B. (2016). Effect of Starter Culture Addition and Processing Method on Proteolysis and Texture Profile of Traditional Dry-Fermented Sausage Petrovská klobása. Int. J. Food Prop..

[4] Molnár H., Kónya É., Zalán Z., Bata-Vidács I., Tömösközi-Farkas R., Székács A., Adányi N. (2018). Chemical characteristics of spice paprika of different origins. Food Control.

[5] Bata-Vidács I., Baka E., Tóth Á., Csernus O., Luzics S., Adányi N., Székács A., Kukolya J. (2018). Investigation of regional differences of the dominant microflora of spice paprika by molecular methods. Food Control.

[6] Enamullah S.M., Rahman A., Sahar N., Haque S.E. (2022). Detection of aflatoxin contamination and incidence of fungi associated with red chili available in local market of Karachi, Pakistan. Pak. J. Bot..

[7] Hadil Mon V., John Kennedy Z., Paranitharan V., Karthikeyan S. (2022). Mycotic contamination and aflatoxin potential of molds in *Capsicum annum* (chili), and chili powder commercialized in south Indian markets. Toxicon.

[8] Chuaysrinule C., Maneeboon T., Roopkham C., Mahakarnchanakul W. (2020). Occurrence of aflatoxin- and ochratoxin A-producing *Aspergillus* species in Thai dried chilli. J. Agric. Food Res..

[9] Chen J., Chen Y., Zhu Q., Wan J. (2023). Ochratoxin A contamination and related high-yield toxin strains in Guizhou dried red chilies. Food Control.

[10] Štursa V., Diviš P., Pořízka J. (2018). Characteristics of paprika samples of different geographical origin. Potravinarstvo.

[11] Zhang Y., Zhu W., Ren H., Tian T., Wang X. (2022). Unraveling the effects of Lactobacillus sakei inoculation on the microbial quality and bacterial community diversity of chili sauce by high-throughput sequencing. Food Sci. Technol..

[12] Zhang Y., Zhu W., Ren H., Tian T., Wang X. (2022). Effects of Lactobacillus sakei inoculation on biogenic amines reduction and nitrite depletion of chili sauce. Food Sci. Technol..

[13] López-Salas D., Oney-Montalvo J.E., Ramírez-Rivera E., Ramírez-Sucre M.O., Rodríguez-Buenfil I.M. (2022). Fermentation of Habanero Pepper by Two Lactic Acid Bacteria and Its Effect on the Production of Volatile Compounds. Fermentation.

[14] Kang S.-Y., Han M.-J. (2005). Effect of kimchi ingredients on the growth of pathogenic and lactic acid bacteria. Korean J. Food Cook. Sci..

[15] Daood H.G., Palotás G., Palotás G., Somogyi G., Pék Z., Helyes L. (2014). Carotenoid and antioxidant content of ground paprika from indoor-cultivated traditional varieties and new hybrids of spice red peppers. Food Res. Int..

[16] Koncsek A., Daood H.G., Horváth Z.H., Fekete M., Véha A., Helyes L. (2019). Improvement of antioxidant content and color stability in spice paprika powder by rosemary extract supplementation. J. Food Process..

[17] Vinković T., Gluščić V., Mendaš G., Vinković Vrček I., Parađiković N., Tkalec M., Štolfa Čamagajevac I. (2018). Phytochemical composition of ground paprika from the eastern Danube region. Poljoprivreda.

[18] Topuz A., Dincer C., Ozdemir K.S., Feng H., Kushad M. (2011). Influence of different drying methods on carotenoids and capsaicinoids of paprika (Cv., Jalapeno). Food Chem..

[19] Cullere M., Novelli E., Dalle Zotte A. (2020). Fat inclusion level, NaCl content and LAB starter cultures in the manufacturing of Italian-type ostrich salami: Weight loss and nutritional traits. Foods.

[20] Das J., Deka M., Gogoi K., Reviews A. (2018). Antimicrobial Activity of Chilli Extracts (*Capsicum chinense*) Against Food Borne Pathogens Escherichia coli and Staphylococcus aureus. Int. J. Res..

[21] Bouarab Chibane L., Degraeve P., Ferhout H., Bouajila J., Oulahal N. (2019). Plant antimicrobial polyphenols as potential natural food preservatives. J. Sci. Food Agric..

[22] Aquilanti L., Santarelli S., Silvestri G., Osimani A., Petruzzelli A., Clementi F. (2007). The microbial ecology of a typical Italian salami during its natural fermentation. Int. J. Food Microbiol..

[23] Tamang J.P., Lama S. (2022). Probiotic properties of yeasts in traditional fermented foods and beverages. J. Appl. Microbiol..

[24] Settanni L., Barbaccia P., Bonanno A., Ponte M., Di Gerlando R., Franciosi E., Di Grigoli A., Gaglio R. (2020). Evolution of indigenous starter microorganisms and physicochemical parameters in spontaneously fermented beef, horse, wild boar and pork salamis produced under controlled conditions. Food Microbiol..

[25] Wang Y., Han J., Wang D., Gao F., Zhang K., Tian J., Jin Y. (2022). Research Update on the Impact of Lactic Acid Bacteria on the Substance Metabolism, Flavor, and Quality Characteristics of Fermented Meat Products. Foods.

[26] Adams M.R. (2001). Why Fermented Foods Can Be Safe.

[27] Wang X., Wang S., Zhao H. (2019). Unraveling microbial community diversity and succession of Chinese Sichuan sausages during spontaneous fermentation by high-throughput sequencing. J. Food Sci. Technol..

[28] Bis-Souza C.V., Penna A.L.B., da Silva Barretto A.C. (2020). Applicability of potentially probiotic Lactobacillus casei in low-fat Italian type salami with added fructooligosaccharides: In Vitro screening and technological evaluation. Meat Sci..

[29] Cebrian E., Nunez F., Alvarez M., Roncero E., Rodriguez M. (2022). Biocontrol of ochratoxigenic Penicillium nordicum in dry-cured fermented sausages by Debaryomyces hansenii and Staphylococcus xylosus. Int. J. Food Microbiol..

[30] Murgia M.A., Marongiu A., Aponte M., Blaiotta G., Deiana P., Mangia N.P. (2019). Impact of a selected Debaryomyces hansenii strain’s inoculation on the quality of Sardinian fermented sausages. Food Res. Int..

[31] Montanari C., Barbieri F., Gardini F., Tabanelli G. (2021). Competition between Starter Cultures and Wild Microbial Population in Sausage Fermentation: A Case Study Regarding a Typical Italian Salami (Ventricina). Foods.

[32] Herrero A.M., Ordonez J.A., de Avila R., Herranz B., de la Hoz L., Cambero M.I. (2007). Breaking strength of dry fermented sausages and their correlation with texture profile analysis (TPA) and physico-chemical characteristics. Meat Sci..

[33] Mathur H., Beresford T.P., Cotter P.D. (2020). Health Benefits of Lactic Acid Bacteria (LAB) Fermentates. Nutrients.

[34] McKELLAR R.C., Knight K.P. (1999). Growth and survival of various strains of enterohemorrhagic Escherichia coli in hydrochloric and acetic acid. J. Food Prot..

[35] Gao Z., Daliri E.B., Wang J., Liu D., Chen S., Ye X., Ding T. (2019). Inhibitory Effect of Lactic Acid Bacteria on Foodborne Pathogens: A Review. J. Food Prot..

[36] Comi G., Andyanto D., Manzano M., Iacumin L. (2016). Lactococcus lactis and Lactobacillus sakei as bio-protective culture to eliminate Leuconostoc mesenteroides spoilage and improve the shelf life and sensorial characteristics of commercial cooked bacon. Food Microbiol..

[37] Blaiotta G., Murru N., Di Cerbo A., Romano R., Aponte M. (2018). Production of probiotic bovine salami using Lactobacillus plantarum 299v as adjunct. J. Sci. Food.

[38] Filipello V., Bonometti E., Campagnani M., Bertoletti I., Romano A., Zuccon F., Campanella C., Losio M.N., Finazzi G. (2020). Investigation and Follow-Up of a Staphylococcal Food Poisoning Outbreak Linked to the Consumption of Traditional Hand-Crafted Alm Cheese. Pathogens.

[39] Bajaj S., Dudeja P. (2019). Food poisoning outbreak in a religious mass gathering. Med. J. Armed Forces India.

[40] Takahashi H., Yokozawa T., Oda T. (2022). Listeria monocytogenes bacteremia one month after contact with raw venison: A case report. IDCases.

[41] Di Gioia D., Mazzola G., Nikodinoska I., Aloisio I., Langerholc T., Rossi M., Raimondi S., Melero B., Rovira J. (2016). Lactic acid bacteria as protective cultures in fermented pork meat to prevent *Clostridium* spp. growth. Int. J. Food Microbiol..

[42] Park Y.S., Lee J.Y. (2012). The effect of kimchi on the microbiological stability of fermented sausage. Meat Sci..

[43] Felföldi Z., Ranga F., Roman I.A., Sestras A.F., Vodnar D.C., Prohens J., Sestras R.E. (2022). Analysis of Physico-Chemical and Organoleptic Fruit Parameters Relevant for Tomato Quality. Agronomy.

[44] Hanganu D. (2020). Research on Enzyme Inhibition Potential and Phenolic Compounds from Origanum *Vulgare* ssp. Vulgare. Farmacia.

[45] Kadar C.B., Paucean A., Simon E., Vodnar D.C., Ranga F., Rusu I.E., Visan V.G., Man S., Chis M.S., Dretcanu G. (2022). Dynamics of Bioactive Compounds during Spontaneous Fermentation of Paste Obtained from *Capsicum* ssp.-Stage towards a Product with Technological Application. Plants.

[46] Socaci S.A., Socaciu C., Mureşan C., Fărcaş A., Tofană M., Vicaş S., Pintea A. (2014). Chemometric discrimination of different tomato cultivars based on their volatile fingerprint in relation to lycopene and total phenolics content. Phytochem. Anal..

[47] Vodnar D.C., Călinoiu L.F., Dulf F.V., Ştefănescu B.E., Crişan G., Socaciu C. (2017). Identification of the bioactive compounds and antioxidant, antimutagenic and antimicrobial activities of thermally processed agro-industrial waste. Food Chem..

[48] Mohd Hassan N., Yusof N.A., Yahaya A.F., Mohd Rozali N.N., Othman R. (2019). Carotenoids of Capsicum Fruits: Pigment Profile and Health-Promoting Functional Attributes. Antioxidants.

[49] Berry H.M., Rickett D.V., Baxter C.J., Enfissi E.M., Fraser P.D. (2019). Carotenoid biosynthesis and sequestration in red chilli pepper fruit and its impact on colour intensity traits. J. Exp. Bot..

[50] Morais H., Rodrigues P., Ramos C., Forgacs E., Cserhati T., Oliveira J. (2002). Effect of ascorbic acid on the stability of beta-carotene and capsanthin in paprika (*Capsicum annuum*) powder. Nahrung.

[51] Markus F., Daood H.G., Kapitany J., Biacs P.A. (1999). Change in the carotenoid and antioxidant content of spice red pepper (paprika) as a function of ripening and some technological factors. J. Agric. Food Chem..

[52] Pereira C., Córdoba M.d.G., Aranda E., Hernández A., Velázquez R., Bartolomé T., Martín A. (2019). Type of paprika as a critical quality factor in Iberian chorizo sausage manufacture. CyTA—J. Food.

[53] Amorim-Carrilho K., Cepeda A., Fente C., Regal P. (2014). Review of methods for analysis of carotenoids. Trends Anal. Chem..

[54] Revilla I., Quintana A.M.V. (2005). The effect of different paprika types on the ripening process and quality of dry sausages. Int. J. Food Sci. Technol..

[55] Eggersdorfer M., Wyss A. (2018). Carotenoids in human nutrition and health. Arch. Biochem. Biophys..

[56] Nishino A., Yasui H., Maoka T. (2016). Reaction of Paprika Carotenoids, Capsanthin and Capsorubin, with Reactive Oxygen Species. J. Agric. Food Chem..

[57] Bosland P.W. (1996). Capsicums: Innovative Uses of an Ancient Crop.

[58] Liu Y., Zhang Z., Hu L. (2022). High efficient freeze-drying technology in food industry. Crit. Rev. Food Sci. Nutr..

[59] Ma Y., Yi J., Jin X., Li X., Feng S., Bi J. (2022). Freeze-Drying of Fruits and Vegetables in Food Industry: Effects on Phytochemicals and Bioactive Properties Attributes—A Comprehensive Review. Food Rev. Int..

[60] Proestos C., Chorianopoulos N., Nychas G.J., Komaitis M. (2005). RP-HPLC analysis of the phenolic compounds of plant extracts. investigation of their antioxidant capacity and antimicrobial activity. J. Agric. Food Chem..

[61] Raccach M. (1984). The antimicrobial activity of phenolic antioxidants in foods: A review 1. J. Food Saf..

[62] Ghasemnezhad M., Sherafati M., Payvast G.A. (2011). Variation in phenolic compounds, ascorbic acid and antioxidant activity of five coloured bell pepper (Capsicum annum) fruits at two different harvest times. J. Funct. Foods.

[63] Antonio A.S., Wiedemann L.S.M., Veiga V.F. (2018). The genus Capsicum: A phytochemical review of bioactive secondary metabolites. Rsc Adv..

[64] De Aguiar A.C., Coutinho J.P., Barbero G.F., Godoy H.T., Martínez J. (2015). Comparative Study of Capsaicinoid Composition in Capsicum Peppers Grown in Brazil. Int. J. Food Prop..

[65] Cvetkovic T., Ranilovic J., Jokic S. (2022). Quality of Pepper Seed By-Products: A Review. Foods.

[66] Bacon K., Boyer R., Denbow C., O’Keefe S., Neilson A., Williams R. (2017). Antibacterial activity of jalapeño pepper (*Capsicum annuum* var. *annuum*) extract fractions against select foodborne pathogens. Food Sci..

[67] Valkova V., Duranova H., Ivanisova E., Galovicova L., Godocikova L., Borotova P., Kunova S., Miklasova K., Lopasovsky L., Mnahoncakova E. (2021). Antioxidant and Antimicrobial Activities of Fruit Extracts from Different Fresh Chili Peppers. Acta Sci. Pol. Technol. Aliment..

[68] Fasolato L., Cardazzo B., Balzan S., Carraro L., Taticchi A., Montemurro F., Novelli E. (2015). Minimum Bactericidal Concentration of Phenols Extracted from Oil Vegetation Water on Spoilers, Starters and Food-Borne Bacteria. Ital. J. Food Saf..

[69] Novelli E., Fasolato L., Cardazzo B., Carraro L., Taticchi A., Balzan S. (2014). Addition of Phenols Compounds to Meat Dough Intended for Salami Manufacture and its Antioxidant Effect. Ital. J. Food Saf..

[70] Ribas-Agustí A., Gratacós-Cubarsí M., Sárraga C., Guàrdia M.D., García-Regueiro J.-A., Castellari M. (2014). Stability of phenolic compounds in dry fermented sausages added with cocoa and grape seed extracts. LWT—Food Sci. Technol..

[71] Zamljen T., Jakopic J., Hudina M., Veberic R., Slatnar A. (2021). Influence of intra and inter species variation in chilies (*Capsicum* spp.) on metabolite composition of three fruit segments. Sci. Rep..

[72] Asami D.K., Hong Y.J., Barrett D.M., Mitchell A.E. (2003). Comparison of the total phenolic and ascorbic acid content of freeze-dried and air-dried marionberry, strawberry, and corn grown using conventional, organic, and sustainable agricultural practices. J. Agric. Food Chem..

[73] Martinez S., Lopez M., Gonzalez-Raurich M., Alvarez A.B. (2005). The effects of ripening stage and processing systems on vitamin C content in sweet peppers (*Capsicum annuum* L.). Int. J. Food Sci. Nutr..

[74] Varvara M., Bozzo G., Celano G., Disanto C., Pagliarone C.N., Celano G.V. (2016). The use of ascorbic acid as a food additive: Technical-legal issues. Ital. J. Food Saf..

[75] Tannenbaum S.R., Wishnok J.S., Leaf C.D. (1991). Inhibition of nitrosamine formation by ascorbic acid. Am. J. Clin. Nutr..

[76] Škrlep M., Ozmec M., Čandek-Potokar M. (2022). Reduced Use of Nitrites and Phosphates in Dry-Fermented Sausages. Processes.

[77] D’Amore T., Russell I., Stewart G.G. (1989). Sugar utilization by yeast during fermentation. J. Ind. Microbiol..

[78] Leroy F., Verluyten J., De Vuyst L. (2006). Functional meat starter cultures for improved sausage fermentation. Int. J. Food Microbiol..

[79] Kollmannsberger H., Rodriguez-Burruezo A., Nitz S., Nuez F. (2011). Volatile and capsaicinoid composition of aji (*Capsicum baccatum*) and rocoto (*Capsicum pubescens*), two *Andean* species of chile peppers. J. Sci. Food Agric..

[80] Pino J., González M., Ceballos L., Centurión-Yah A.R., Trujillo-Aguirre J., Latournerie-Moreno L., Sauri-Duch E. (2007). Characterization of total capsaicinoids, colour and volatile compounds of Habanero chilli pepper (*Capsicum chinense* Jack.) cultivars grown in Yucatan. Food Chem..

[81] Thierry A., Richoux R., Kerjean J.-R. (2004). Isovaleric acid is mainly produced by Propionibacterium freudenreichii in Swiss cheese. Int. Dairy J..

[82] Shim S.-M., Kim J.Y., Lee S.M., Park J.-B., Oh S.-K., Kim Y.-S. (2012). Profiling of fermentative metabolites in kimchi: Volatile and non-volatile organic acids. J. Korean Soc. Appl. Biol. Chem..

[83] Bozkurt H., Erkmen O. (2005). Effects of salt, starter culture and production techniques on the quality of hot pepper paste. J. Food Eng..

[84] Piasecka-Jóźwiak K., Rozmierska J., Chablowska B., Stecka K.M., Skapska S., Kliszcz M., Szkudzinska–Rzeszowiak E. (2013). Starter cultures for lactic acid fermentation of sweet pepper, pattypan squash and tomatoes. Pol. J. Food Nutr. Sci..

[85] Alberto M.R., Perera M.F., Arena M.E. (2013). Lactic acid fermentation of peppers. Food Nutr. Sci..

[86] Dias I., Laranjo M., Potes M.E., Agulheiro-Santos A.C., Ricardo-Rodrigues S., Fialho A.R., Vestia J., Fraqueza M.J., Oliveira M., Elias M. (2020). Autochthonous Starter Cultures Are Able to Reduce Biogenic Amines in a Traditional Portuguese Smoked Fermented Sausage. Microorganisms.

[87] Gardner N.J., Savard T., Obermeier P., Caldwell G., Champagne C.P. (2001). Selection and characterization of mixed starter cultures for lactic acid fermentation of carrot, cabbage, beet and onion vegetable mixtures. Int. J. Food Microbiol..

[88] Pederson C.S., Albury M.N. (1969). The Sauerkraut Fermentation.

[89] Cocolin L., Urso R., Rantsiou K., Cantoni C., Comi G. (2006). Dynamics and characterization of yeasts during natural fermentation of Italian sausages. FEMS Yeast Res..

[90] Gonzalez-Fernandez C., Santos E.M., Rovira J., Jaime I. (2006). The effect of sugar concentration and starter culture on instrumental and sensory textural properties of chorizo-Spanish dry-cured sausage. Meat Sci..

[91] Roca M., Incze K. (1990). Fermented sausages. Food Rev. Int..

[92] Csillag P., Török Á. (2019). PDO Kalocsai paprika powder in Hungary. Sustainability of European Food Quality Schemes.

