# Supplementary material for: Freeze-Dried Powder of Fermented Chili Paste—New Approach to Cured Salami Production
Source: Foods. 2022 Nov 18;11(22):3716. doi: 10.3390/foods11223716 (PMC9689597; doi:10.3390/foods11223716)
Supplement: Supplementary file 1 [file foods-11-03716-s001.zip › foods-2016435-supplementary.pdf]

foods

MDPI

Supplementary Materials

Freeze-Dried Powder of Fermented Chili Paste—New Approach to Cured Salami Production

Adriana Păucean [1,*,†], Csaba Balázs Kádár [1,†], Elemér Simon [2], Dan Cristian Vodnar [2], Floricuța Ranga [2], Iulian Eugen Rusu [1], Vasile-Gheorghe Vișan [3], Sonia-Ancuța Socaci [2], Simona Man [1], Maria Simona Chiș [1], Anamaria Pop [1], Anda E. Tanislav [1] and Vlad Mureșan [1]

[1] Department of Food Engineering, Faculty of Food Science and Technology, University of Agricultural Sciences and Veterinary Medicine, 3–5 Mănăștur Street, 400372 Cluj-Napoca, Romania
[2] Department of Food Science, Faculty of Food Science and Technology, University of Agricultural Science and Veterinary Medicine, 3–5 Calea Mănăstur, 400372 Cluj-Napoca, Romania
[3] Department of Fundamental Sciences, Faculty of Animal Science and Biotechnologies, University of Agricultural Sciences and Veterinary Medicine, 3–5 Mănăștur Street, 400372 Cluj-Napoca, Romania
* Correspondence: adriana.paucean@usamvcluj.ro
† These authors contributed equally to this work.

Table S1. Variation of aroma compounds in the examined samples of fresh and fermented chilies (%) (Values (%) represent means for 3 replicates; n.i.—not identified).

	Cherry		Cayenne		Fatalii		Habanero	
Volatile compounds identified	fresh (%)	fermented (%)	fresh (%)	fermented (%)	fresh (%)	fermented (%)	fresh (%)	fermented (%)
1,3,6-Octatriene, 3,7-dimethyl-, (Z)-	0.21	0.60		0.34	0.23	0.29	0.03	0.08
1,3-Hexadiene, 3-ethyl-2-methyl-	2.54		2.2		0.01	0.01	0.09	
.beta.-Linalool		1.33		12.63				
1,6-Octadiene, 2,6-dimethyl-					1.42	2.72		
1-Butanol, 2-methyl-					0.15	0.42		0.10
1-Butanol, 3-methyl-	1.03	6.59		2.07	0.4	1.29	0.14	0.29
1-Heptanol					0.09	0.16	0.03	
1-Hexanol	5.58	8.61	8.72	14.87	0.73	1.63	0.72	0.57
1-Hexanol, 4-methyl-							0.06	
1-Hexanol, 4-methyl-, (S)-						0.03		
1-Nonanol					0.07	0.14		
1-Octanol						0.01		
1-Pentanol	0.68				0.13	0.49	0.17	0.04
1-Pentanol, 2-methyl					0.12			
1-Pentanol, 4-methyl-	4.75	7.36	2.98	1.11	2.64	6.06	5.68	4.97
2-Butanol, 3-methyl-, acetate					2.64			
2-Heptenal, (Z)-	0.26	0.26		0.87				
2-Hexen-1-ol, (E)-	12.98	26.06	34.95	30.87	1.08	1.24	0.64	2.51
2-Hexen-1-ol, acetate		6.18		4.42		0.28		0.53
2-Hexenal	40.42	0.80	30.06	3.24	1.12		1.92	
2-Nonanone					0.02			
2-Nonenal, (E)-	0.29							
2-Octenal, (E)-	1.12	0.29	0.37					

2-Pentanol						4.40		
2-Pentanol, acetate						0.08		
3-Heptanone, 5-ethyl-4-methyl-					0.03			
3-Hexen-1-ol					0.54			
3-Hexen-1-ol, (Z)-						1.26	0.72	1.59
3-Hexen-1-ol, acetate, (Z)-						0.19		0.19
3-Pentanol								0.05
3-Pentanone, 2-methyl-						0.06		0.02
3-Penten-1-ol, 4-methyl-						0.08		
Acetic acid, 2-methylpropyl ester					0.11	0.23		
Acetic acid, heptyl ester						0.60		0.41
Acetic acid, hexyl ester		3.85		3.98	0.11	0.72	0.06	0.79
Acetic acid, nonyl ester						0.08		
Acetophenone	0.73	1.36	4.06	3.98	0.05	0.04	0.11	0.12
Benzaldehyde	0.72	1.52	2.64	1.94	0.01	0.02	0.05	0.07
Benzeneacetaldehyde	0.24	0.85		0.60		0.01		0.02
Benzoic Acid			1.65	0.46		0.03		0.11
Butanoic acid, 2-methyl-, 2-methylbutyl ester					0.02	0.03	0.03	0.04
Butanoic acid, 2-methyl-, 2-methylpropyl ester					0.14	0.12		0.03
Butanoic acid, 2-methyl-, 3-methylbutyl ester	0.44				0.20	0.31	0.16	
Butanoic acid, 2-methyl-, ethyl ester						0.17		0.29
Butanoic acid, 2-methyl-, hexyl ester	4.56	5.24	4.02	1.52	10.7	10.93	13.16	12.3
Butanoic acid, 2-methyl-, methyl ester, (.+/-.)-					0.12			0.15
Butanoic acid, 2-methyl-, pentyl ester					0.24	0.21	0.18	0.16
Butanoic acid, 2-methyl-, propyl ester					0.05	0.02		
Butanoic acid, 2-methylbutyl ester					0.05			
Butanoic acid, 3-hexenyl ester, (E)-							0.32	
Butanoic acid, 3-hexenyl ester, (Z)-					0.71	0.57		0.20
Butanoic acid, 3-methyl-, 2-methylpropyl ester					0.88	0.80	0.13	0.12
Butanoic acid, 3-methyl-, 3-methylbutyl ester		0.57			2.35	2.27	0.90	0.80
Butanoic acid, 3-methyl-, hexyl ester	5.81	6.61	3.37	0.91	36.27	36.36	46.58	47.29
n.i							7.59	
Butanoic acid, 3-methyl-, propyl ester					0.06	0.02		

Butanoic acid, hexyl ester		0.90			5.55	5.44	0.04	0.51
cis-3-Hexenyl isovalerate			0.56				11.55	8.93
cis-3-Hexenyl-.alpha.-methylbutyrate					2.41	1.81	2.19	1.39
Cyclohexane, 1,1,3-trimethyl-					0.23	1.92		0.22
beta.-Elemene	1.29							
Disulfide, dimethyl				0.70				
D-Limonene				0.51				
Cuminone	8.70							
Furan, 2-pentyl-	0.55	1.56	1.35	2.45			0.03	0.08
Heptanal	0.30							
Hexanal	2.94	4.80	0.51	3.09		0.03	0.06	
Hexanoic acid, 2-methylbutyl ester		3.22						
Hexanoic acid, 2-methylpropyl ester					0.02	0.03		
Hexanoic acid, 2-propenyl ester						0.33		
Hexanoic acid, ethyl ester		0.55		0.91				0.13
Hexanoic acid, hexyl ester	1.70	1.11						
Hexanoic acid, methyl ester						0.04		
Hexanoic acid, propyl ester			0.60					
Hexyl n-valerate		0.64	0.26		13.99			6.79
Methyl Salicylate			0.94				0.11	0.25
n-Amyl isovalerate					1.30	1.29	0.87	0.85
Nonanal	1.10							
n-Propyl acetate		4.54		9.11	0.06	0.12		
n-Valeric acid cis-3-hexenyl ester							0.09	0.06
Octanoic acid, methyl ester						0.03		
Pentanal			0.76	0.93				
Pentanoic acid, 1,1-dimethylpropyl ester						0.05		
Pentanoic acid, 2-pentyl ester								0.08
Pentanoic acid, 4-methyl-, ethyl ester						0.15		0.20
Pentanoic acid, 4-methyl-, methyl ester						0.21		0.05
Pentanoic acid, 5-hexen-1-yl ester		0.81						
Pentanoic acid, cyclohexyl ester					13.43	11.48		
Pentanoic acid, pentyl ester								0.29
Phenol, 2-methoxy-						0.30		0.05
Phenol, 2-methoxy-4-methyl-		2.43		0.40		0.44		1.46
Propanoic acid, 2-hydroxy-, ethyl ester, (S)-				0.51				
Propanoic acid, 2-hydroxy-2-methyl-						0.08		

Propanoic acid, 2-methyl-, 2-methylpropyl ester					0.12			
Propanoic acid, 2-methyl-, ethyl ester						0.07		0.03
Propanoic acid, 2-methyl-, heptyl ester							0.11	0.10
Propanoic acid, 2-methyl-, hexyl ester	1.06	1.37			1.81	1.71	5.45	4.69
Propanoic acid, 2-methyl-, pentyl ester					0.08	0.09	0.03	
Propanoic acid, 2-methyl-, propyl ester					0.06			
